# Synthesis and Chiral Separation of Fibratol, Isopropyl 2-(4-((4-chlorophenyl)(hydroxyl) methyl)-phenoxy)-2-methylpropanoate

**DOI:** 10.4236/ijoc.2018.82015

**Published:** 2018-05-30

**Authors:** Amanda E. Kotheimer, Wahajul Haq, Ganesaratnam K. Balendiran

**Affiliations:** 1 Department of Chemistry, Youngstown State University, One University Plaza, Youngstown, OH, USA; 2 Medicinal Chemistry Division, Central Drug Research Institute, Lucknow, India

**Keywords:** Reduction, Chirality, Optical Activity, Fibrate

## Abstract

Practical synthetic route for the formation of enantiomeric mixture of Isopropyl 2-(4-((4-chlorophenyl)(hydroxyl)methyl)phenoxy)-2-methylpropanoate (Fibratol 2a/b) from isopropyl 2-(4-(4-chlorobenzoyl)phenoxy)-2-methylpropanoate (Fenofibrate 1) has been developed. Method has also been established for the chiral separation of enantiomers of Fibratol 2a/b that is synthesized using the route mentioned above. The optical activity determined for enantiomerically separated Fibratol (2a) and Fibratol (2b) are −5.2° and 8.0° which reflect their ability to rotate plane polarized light counterclockwise (*levo*) and clockwise (*dextro*), respectively.

## 1. Introduction

Rentsch [1] reported that about 56% of the synthetic drugs currently in use are chiral compounds. Though 88% of these chiral synthetic drugs are used therapeutically as racemates the recent trend in industry is to market the drugs in a pure enantiomeric form to give new life to old drugs for variety of reasons [1]. The leading single enantiomer blockbuster drugs (along with their corresponding medical/clinical application) are: Atorvastatin calcium (cardiovascular); Simvastatin (cardiovascular); Pravastatin sodium (cardiovascular); Paroxetine hydrochloride (CNS); Clopidogrel bisulfate (hematology); Sertraline hydrochloride (CNS); Fluticasone propionate and Salmeterol xinafoate (respiratory); Esomeprazole magnesium (gastrointestinal); Amoxicillin and Potassium clavulanate (antibiotic); and Valsartan (cardiovascular) [2] to mention a few.

Fenofibrate has shown inhibition properties towards Aldo-Keto reductase protein family members, Aldose Reductase and AKR1B10 recently [3] [4]. However, fibrates were previously believed to be ligands for the nuclear receptor, PPAR*α* (peroxisome proliferator-activated receptor *α*) and are consequently used as therapeutic agents in the treatment of hyperlipidemia, heart disease and diabetic complications [5] [6] [7]. We describe herein methods to generate alcoholic/hydroxy derivatives of the above compounds, the enantiomeric separation and determine their chirality.

## 2. Experimental

General Chemicals, Procedures and Instruments. The reaction was conducted at room temperature, unless otherwise noted [8]. Purification was accomplished by column chromatography, or high performance liquid chromatography. The ^1^H and ^13^C NMR were recorded on a Bruker Advance II 400 MHz NMR spectrometer with an indirect detection probe. Chemical shifts were reported in parts per million (ppm) from a standard of tetramethylsilane (TMS) in CDCl_3_ (0.1% w/v TMS). Signals in NMR spectra are defined as follows: s (singlet), d (doublet), t (triplet), m (multiplet), dd (doublet of doublets), pd (pseudo doublet), and all coupling constants (J) are labeled in Hertz. The mass spectra (MS) reported were obtained on a Bruker Esquire-HP LC/MS spectrometer in ESI+ detection mode. Samples were dissolved in methanol at a concentration of 1 mg/mL. Thin layer chromatography (TLC) was performed on oven dried Whatman aluminum-backed plates with varying eluent systems. Flash column chromatography was performed using oven dried 32 – 60 mesh 60-Å silica gel with varying eluent systems. A Perkin-Elmer 343 polarimeter was used to measure the optical rotation of all homogenous compounds. Infrared spectra were taken on a Thermo Electron Corporation IR 200 spectrophotometer and analyzed using EZ-OMNIC software.

### 2.1. Synthesis of Isopropyl 2-(4-((4-chlorophenyl)(hydroxyl)methyl)phenoxy)-2-methylpropanoate (Fibratol 2a/b)

To an oven-dried, 100 mL round bottomed flask, fitted with magnetic stir-bar, 0.425 g (1.18 mmol) of Fenofibrate (1) was added and then dissolved in 16 mL of methanol. When partially dissolved, an ice water bath was placed under the reaction flask and 0.093 g (2.46 mmol) of NaBH_4_ was added slowly in small portions. The ice water bath was removed and the solution was allowed to stir at room temperature (24°C) for 3 hours. The reaction was monitored by TLC (7:3 ethyl acetate/hexane) and it resulted in two spots with corresponding Rf values of 0.73 and 0.77. The reaction mixture was placed in an ice water bath and 15 mL of chilled 5% HCl was added very slowly. The crude reaction mixture was poured onto 10 mL water, separated, extracted with ethyl acetate (2 × 15 mL) and dried over MgSO_4_. Using a Whatman #1 filter pad, the reaction mixture was filtered and the solvent was removed in vacuo resulting in a clear oil with 66.7% yield. Fibratol (2a/2b) has the following spectroscopic properties: ^1^H NMR: δ 1.21 (d, 6H, ^3^J= 5.76 Hz), 1.57 (s, 6H), 2.16 (s, 2H), 5.07 (septet, 1H, ^3^J = 6.31 Hz), 5.76 (s, 2H), 6.79 (pd (FF’ part of FF’HH’ pattern) ^3^J = 9.12 Hz, 2H), 7.19 (pd (GG’ part of GG’II’ pattern) ^3^J = 8.84 Hz, 2H), 7.29 (pd (HH’ part of HH’FF’ pattern and (pd (II’ part of II’GG’ pattern) ^3^J = 8.79 Hz, 4H); ^13^C NMR: δ 21.53, 25.39, 69.35, 79.47, 117.31, 128.55, 130.28, 131.16, 131.94, 136.47, 138.37, 159.77, 173.10, 194.25; ESI+ m/z (calculated by ACDLab2014): 362.8; m/z (experimental/found): 361.2 + 23 (385.2); IR spectrum (cm^−1^): 3466, 2985, 2939, 2876, 1904, 1728, 1606, 1505, 1465, 1381, 1096, 1012, 972, 827, 718, 684, 618.

### 2.2. Chiral Separation of Enantiomeric Products

High Performance liquid chromatography (HPLC) analyses were performed on a Waters 1525 Binary HPLC pump coupled to a Waters 2487 dual λ absorbance detector using Breeze software. All solvents were degassed for 1 hr under helium. A Lux Amylose-2 chiral column (5 μm, 4.6 × 250 mm) was selected for the separation of chiral compounds that had a unique selector amylose tris(5-chloro-2-methylphenylcarbamate). Reduced racemic fenofibrate was separated by chiral separations techniques. A 5 mg of reaction product was dissolved in 1.5 mL of HPLC grade methanol. A 20 μL of this solution was injected by a 250 μL syringe at a flow rate of 1 mL/min and analyzed using HPLC by monitoring the flow through at 275 nm.

Specific rotation [*α*], reflects the magnitude of the rotation of each enantiomer. In addition the optical rotation is dependent on the wavelength of light used, temperature, solvent, concentration and the length of the polarimeter tube/cell. When monochromatic light from a sodium lamp D-line at 589 nm is used as the source, *α* can be defined by (Equation (1)) as

(1) $${\left[\alpha \right]}_{D}^{25}\;=\;\left\{\text{observed rotation}\left(\text{degrees}\right)/\text{length of sample tube}(dm)\times \text{concentration}\left(\frac{g}{ml}\right)\right\}$$

where T (=25°C) is the measurement temperature, λ is the wavelength of light employed, *α* is the observed rotation, l is the path length and c is the concentration in grams per mL (the density of the pure substances) or grams per 100 mL. Specific rotation may also be expressed as degrees per mole of the substance where the conditions of measurement (*i.e.* solvent used, light source and path length) are also specified [9].

## 3. Results and Discussions

**Scheme 1:**
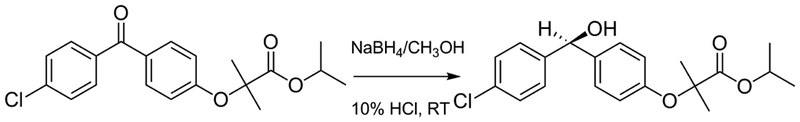
Formation of Fibratol (2a/b) from Fenofibrate (1)

Primary and secondary alcohols can be synthesized by reduction of the corresponding carbonyl compounds using a great variety of reagents [10]. Though ketones can be reduced by a wide variety of reagents, sodium borohydride (NaBH_4_) is considered to be the most useful one. The current reaction conditions (Scheme 1) were chosen because: 1) the reducing reagent, NaBH_4_, is inexpensive and readily available from commercial sources; 2) NaBH_4_ is more selective than any other hydride source because it reduces ketones but not carboxylic acids/esters; 3) the reaction can be maintained (tightly controlled) since it is conducted at room temperature (RT) and (iv) it is a one step reaction.

### 3.1. Synthesis of Fibratol (2a/2b)

The reduction (Scheme 1) of Fenofibrate (1), to Fibratol (2a/2b) was monitored via TLC (7:3 ethyl acetate/hexane v:v) until complete conversion of (1) to the final product (2a/2b) occur. Completion of the reaction after 3 hours is supported by 1) the shift of the -OH signal at 5.76 ppm in ^1^H NMR; 2) presence of an experimental m/z peak at 361.2, corresponding to the calculated m/z at 362.8 in MS; 3) appearance of an absorbance band at 3466 cm^−1^ in the IR spectra and 4) the disappearance of the signal at 194.0 ppm and appearance of 74.9 ppm peak in ^13^C NMR and further indicate the absence of the ketone carbonyl and the presence of OH group in the product.

### 3.2. Chiral Separation of Fibratol (2a/2b)

**Figure 1.**
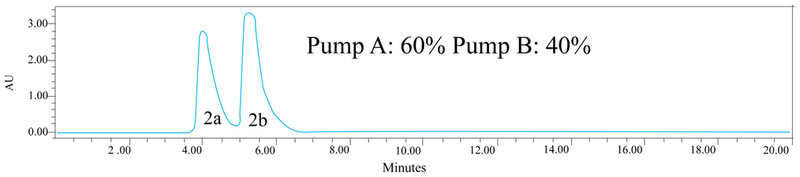
Chiral separation of racemic Fibratol (2a/2b) without the use of any gradient method. Plot of Absorbance (AU) against Retention parameter (time) in min of the enantiomers are shown. Mobile phase 1 and 2 are delivered through Pump A and B, respectively.

HPLC technique was selected for the enantiomeric separation of Fibratol (2a/b). The mobile phase 1 consisted of 500 mL of acetonitrile and 0.5 mL of diethylamine and mobile phase 2 comprised of 500 mL of isopropanol and 0.5 mL of diethylamine. For 20 μL sample size, the run time of 20 minutes at 275 nm with constant flow rate at 1 mL/min was used. Corresponding retention times for separation of each enantiomer are shown in Table 1 and Figure 1.

The difference in the retention time between peaks 2a and 2b increases from trial 1 to 4. Moreover trial 4 gave better resolved peaks for 2a and 2b compared to trial 1. Although there are minor differences in their retention times when varying the solvent systems, 60% of mobile phase 1 and 40% of mobile phase 2 yielded the best separation of the peaks.

### 3.3. Optical Activity

The angle of rotation, *α*, of the plane of polarized light by an optically active forms of Fibratol (2a/2b) were measured by polarimetry technique. A 100 mg of Fibratol (2a) and Fibratol (2b) that were separated by chiral column were dissolved individually in 2 mL of methanol. The optical activity of Fibratol (2a) and Fibratol (2b) were −5.2° reflecting counterclockwise (levo) and 8.0° suggesting clockwise (dextro) rotation of plane polarized light, respectively. The trend demonstrated in resolving the chiral isomers of fibratol under otherwise identical condition (method, column, rate, pressure) reflects the amount of mobile phase 1 and 2 used in trials.

## 4. Conclusion

In conclusion, we have developed practical synthetic route to generate reduced derivatives of fenofibrate. This method is expected to be useful in converting ketone moiety of fenofibrate to its alcohol. This alcohol of fenofibrate will have chiral C atom. Molecular chirality is a fundamental consideration in drug discovery as it plays a critical role in therapeutics. Living organisms often display different biological responses to drug enantiomers when they are treated separately. Systematically it is customary for one enantiomer of a molecule to be biologically active while the other enantiomer to exist chemically toxic to the living systems. As a result, many pharmaceutical creations have taken advantage of this phenomenon and today the majority of drugs in the market are of a single-enantiomer.

## Figures and Tables

**Table 1.** HPLC separation of racemic Fibratol (2a/2b) without any gradient but with varying amounts of % A (Pump A-mobile phase 1) and % B (Pump B-mobile phase 2).

Trial	% A	% B	Retention Time (min.)	
			for 2a	for 2b
1	95	5	4.155	5.142
2	80	20	4.145	5.12
3	70	30	4.109	5.181
4	60	40	3.936	5.046

## Abbreviations and Acronyms

CNS: Central nervous system
J: Coupling constants
d: Doublet
dd: Doublet of doublets
MS: Mass spectra
m: Multiplet
ppm: Parts per million
pd: Pseudo doublet
RT: Room temperature
s: Singlet
TLC: Thin layer chromatography
TMS: Tertramethylsilane
t: Triplet
